# TAGAP expression influences CD4+ T cell differentiation, immune infiltration, and cytotoxicity in LUAD through the STAT pathway: implications for immunotherapy

**DOI:** 10.3389/fimmu.2023.1224340

**Published:** 2023-09-06

**Authors:** Zhanyu Xu, Tiaozhan Zheng, Zhiwen Zheng, Wei Jiang, Liuliu Huang, Kun Deng, Liqiang Yuan, Fanglu Qin, Yu Sun, Junqi Qin, Shikang Li

**Affiliations:** ^1^ Department of Thoracic and Cardiovascular Surgery, The First Affiliated Hospital of Guangxi Medical University, Nanning, Guangxi Zhuang Autonomous Region, China; ^2^ School of Information and Management, Guangxi Medical University, Nanning, Guangxi Zhuang Autonomous Region, China

**Keywords:** TAGAP, CD4+ T cells, lung adenocarcinoma (LUAD), JAK-STAT signaling pathway, immunotherapy

## Abstract

**Background:**

T-cell Activation GTPase Activating Protein (TAGAP) plays a role in immune cell regulation. This study aimed to investigate TAGAP’s expression and its potential impact on CD4+ T cell function and prognosis in lung adenocarcinoma (LUAD).

**Methods:**

We analyzed TAGAP expression and its correlation with immune infiltration and clinical data in LUAD patients using multiple datasets, including The Cancer Genome Atlas (TCGA-LUAD), Gene Expression Omnibus (GEO), and scRNA-seq datasets. *In vitro* and *in vivo* experiments were conducted to explore the role of TAGAP in CD4+ T cell function, chemotaxis, and cytotoxicity.

**Results:**

TAGAP expression was significantly lower in LUAD tissues compared to normal tissues, and high TAGAP expression correlated with better prognosis in LUAD patients. TAGAP was positively correlated with immune/stromal/ESTIMATE scores and immune cell infiltration in LUAD. Single-cell RNA sequencing revealed that TAGAP was primarily distributed in CD4+/CD8+ T cells. *In vitro* experiments showed that TAGAP overexpression enhanced CD4+ T cell cytotoxicity, proliferation, and chemotaxis. Gene Set Enrichment Analysis (GSEA) indicated that TAGAP was enriched in the JAK-STAT signaling pathway. *In vivo* experiments in a xenograft tumor model demonstrated that TAGAP overexpression suppressed tumor growth and promoted CD4+ T cell cytotoxicity.

**Conclusions:**

TAGAP influences CD4+ T cell differentiation and function in LUAD through the STAT pathway, promoting immune infiltration and cytotoxicity. This study provides a scientific basis for developing novel LUAD immunotherapy strategies and exploring new therapeutic targets.

## 1 Introduction

Lung cancer is the leading cause of cancer-related deaths worldwide, with lung adenocarcinoma (LUAD) accounting for nearly 50% of all cases and a 5-year overall survival (OS) rate still below 20% (1, 2). In recent years, immunotherapy has become an established strategy in cancer treatment, including LUAD (3). The main difference between immunotherapy and traditional radio-chemotherapy or molecular targeted therapy is that immunotherapy can provide long-term and stable therapeutic effects for some patients, greatly improving their prognosis and quality of life (4). However, tumor heterogeneity and individual differences limit its practical application, as immune therapy benefits only a small group of patients due to the suppression of immune effects in some tumor microenvironments and the occurrence of drug resistance and adverse reactions (5).

Most antitumor immunotherapies focus on stimulating cytotoxic T lymphocytes (CTLs), the main T cell group that kills tumors. Current research suggests that activating CD8+ T cells is the primary way of antitumor immunity, while CD4+ T cells are also essential (6). In the context of cancer, multiple lines of evidence highlight the importance of CD4+ T cell recognition of tumor antigens for cancer immunotherapy responses. CD4+ T cells have complex immunoregulatory functions, with different subtypes exerting opposing effects on cancer progression (7, 8). However, research on CD4+ T cell differentiation and tumor regulation in LUAD tumor microenvironment is still insufficient.

Rho GTPases act as molecular switches in various cellular processes by transitioning between GTP-bound “on state” and GDP-bound “off state” (9). Rho GTPases reportedly coordinate the biochemical or cytoskeletal pathways that cause T cells to polarize towards antigen-presenting cells (APCs), and disruption of these pathways interferes with T cell responses to stimulation and affects effector T cell differentiation (10). The functions of most Rho GTPase-activating proteins (RhoGAPs) in T cells remain largely unexplored. The T cell-activated Rho GTPase activating protein (TAGAP) encodes a member of the RhoGAP superfamily, which participates in the Rho GTPase cycle.

TAGAP is highly expressed in the immune system, including T and B lymphocytes, dendritic cells, and natural killer cells, but mainly transiently expressed in activated T lymphocytes (11). As a GTPase-activating protein, TAGAP may be involved in T cell activation through its interaction with IL2, affecting the development of autoimmune diseases (12). TAGAP has been shown to be associated with immune cell infiltration in various cancers. In LUAD-related research, TAGAP was found to be a key gene affecting the tumor microenvironment and closely related to LUAD prognosis (13). However, the role of TAGAP in the progression of malignant tumors remains unclear.

In this study, we first analyzed the expression of TAGAP in LUAD patient tumor tissue samples using bioinformatics and collected corresponding tissue samples. We further investigated the essential role of TAGAP in CD4+ T cell immunoregulation through functional cellular experiments and validated that TAGAP overexpression promotes CD4+ T cell immune infiltration and cytotoxicity, slowing LUAD growth in animal models. Mechanistically, we preliminarily examined the JAK-STAT pathway-related proteins in CD4+ T cells with TAGAP overexpression and found that TAGAP overexpression activates the STAT pathway. In summary, our research provides initial insights into how TAGAP affects CD4+ T cell differentiation and function within the tumor microenvironment through the STAT pathway, thereby inhibiting LUAD malignancy progression. This study offers a scientific basis for developing novel LUAD immunotherapy strategies and exploring new therapeutic targets.

## 2 Materials and methods

### 2.1 Datasets and bioinformatics analyses

The RNA-seq expression, somatic mutation data, and clinical data of lung cancer patients were retrieved from The Cancer Genome Atlas (TCGA) (http://cancergenome.nih.gov/) (14). 535 LUAD samples were divided into high and low expression groups based on the median TAGAP expression. The “Limma” R package was used for differential expression analysis of RNA-seq data (15). The relationship between mRNA expression levels and overall survival (OS) of LUAD patients were analyzed using the “survival” R package. Gene expression profiling interaction analysis (GEPIA) was used for TAGAP survival and immune cell signature correlation analyses (16).

### 2.2 Immune infiltrates analysis

The high and low immune groups with median cutoff were studied with the “CIBERSORT” and “ESTIMATE” algorithms in TCGA samples to assess TAGAP expression levels in immune cells. CIBERSORT is based on a deconvolution algorithm that can be used to quantify 22 immune cell types.

The single cell RNA sequencing data (GSE99254) was downloaded from Gene Expression Omnibus (GEO; https://www.ncbi.nlm.nih.gov/). LUAD case samples were selected from the matrix for further analyses. A total of 9553 single-cell RNA sequencing samples (1,894 normal, 4274 tumor, and 3,385 peripheral blood samples) were collected from 11 LUAD cases. We used the “Seurat V4.0.4” R package to process the single-cell data expression matrix. First, “CreateSeuratObject” was used to screen gene expression data, “NormalizeData” to normalize gene expression data, and “FindVariableGenes” to confirm 2000 highly variable genes. Then, the principal component analysis (PCA) was performed using “RunPCA”. The PCs that could be used were screened by the “FJackStraw” function and grouped with “Find Clusters” at the highest resolution. Finally, “tSNA” was used for visualization. Moreover, we used the “Single R” and “celldex” R packages through “HumanPrimaryCellAtlasData” for the cell to perform the annotation. “Feature Plot” and “Vln Plot” were used to visualize TAGAP expression.

Tumor Immune Dysfunction and Exclusion (TIDE, http://tide.dfci.harvard.edu/) was utilized to investigate the relationship between TAGAP expression and immune cells, as well as to calculate immunotherapy response prediction (17). GEO datasets were included if the following criteria were met: the source of the dataset samples was non-small cell lung cancer or lung adenocarcinoma tissue specimens, each of which had a clear pathological diagnosis; and the dataset type was microarray or high-throughput sequencing data. Exclusion criteria: data analysis required the removal of all data in each dataset except for lung adenocarcinoma.

### 2.3 Gene set enrichment analysis

GSEA is a reliable molecular spectrum data analysis tool. It links previous knowledge with newly generated data and uses overlapping statistics to reveal gene behavior in health and disease states (18). In the present study, we used the GSEA 4.1.0 software to perform analyses based on the TAGAP expression matrix. The median of TAGAP expression was the cut-off value, divided into high- and low-expression groups. A thousand genome permutations were analyzed. We used *p*-value and normalized enrichment score (NES) to classify enriched pathways of each phenotype, and evaluate the correlation between TAGAP expression and immune expression-related factors.

### 2.4 Sample collection

From July 2020 to January 2021, 26 LUAD tissue samples were acquired from the Guangxi Medical University’s First Affiliated Hospital by surgical excision and preserved at -80°C. Two independent pathologists verified the LUAD diagnosis. The ethics committee of the Guangxi Medical University’s First Affiliated Hospital gave approved this investigation (2020-KY-NSFC-074).

### 2.5 Quantitative real-time polymerase chain reaction

We used qRT-PCR to predict the correlation between TAGAP and CD4 and CD8 expressions. After total RNA extraction, cDNA synthesis was performed using random hmers and SuperScript III reverse transcriptase for qRT-PCR. The results were estimated by the 2-Δ method. A *p* < 0.05 was considered statistically significant. The primer sequences were as follows: TAGAP Forward Sequence 5’-3’: GACAGACTTGAAAGCATCGC and Reverse Sequence 5’-3’: CTCCTGAATATCCCTTCCGTTG; CD8 Forward Sequence 5’-3’: GGACTTCGCCTGTGATATCTAC and Reverse Sequence 5’-3’: TTGTCTCCCGATTTGACCAC; CD4 Forward Sequence 5’-3’: GCCCTTGAAGCGAAAACAG and Reverse Sequence 5’-3’: CTCCTTGTTCTCCAGTTTCAAAC; GAPDH Forward Sequence 5’-3’: ACATCGCTCAGACACCATG and Reverse Sequence 5’-3’: TGTAGTTGAGGTCAATGAAGGG. GAPDH was used as an internal control.

### 2.6 Plasmid construction, cell transfection, and efficiency verification

The coding sequence (CDS) region of TAGAP was obtained by querying the NCBI website (https://www.ncbi.nlm.nih.gov/nuccore/NM_152133.3). Based on the sequence, the CDS region of TAGAP was inserted into the cloning vector LV5 (EF-1a/GFP&Puro), with the restriction enzyme sites NotI and BamHI used for digestion. The overexpression plasmid of TAGAP was successfully constructed, and sequencing and comparative analysis of the vector confirmed that the gene sequence matched the intended sequence, verifying the successful construction of the vector.

CD4 T cells were seeded in a 24-well plate and transfected with lentivirus carrying the expression vectors LV-TAGAP and LV-NC. After transfection, cells were collected, and RNA was extracted using the Trizol method. The RNA was dissolved in 30 μL of DEPC-treated water, and its quality was assessed using Nanodrop. Qualified RNA samples were stored at -80°C. The 2^-^ΔΔCt method was employed to calculate the relative gene expression normalized to GAPDH.

### 2.7 Cell culture

Peripheral blood mononuclear cells (hPBMC) from healthy individuals were purchased from ATCC (PCS-800-011). The Dynabeads^®^ CD4 Positive Isolation Kit (cat. 11331D, Invitrogen™) was used for cell sorting, and Dynabeads™ Human T-Activator CD3/CD28/CD137 (cat. 11163D, Gibco™) were used to activate CD4 T cells.

### 2.8 Cell proliferation

CD4 T cells transfected with LV-NC and LV-TAGAP were collected and resuspended. The cell suspension was seeded in a 96-well plate at a density of 2000 cells per well in 90 μL of culture medium. The plate was incubated at 37°C with 5% CO2. On days 0, 1, 3, 5, and 7, the absorbance of each well was measured at 490 nm using an MTT assay kit.

### 2.9 Cell cytotoxicity assay

A549 cells are a human lung adenocarcinoma cell line, commonly used in non-small cell lung cancer research for its pathological relevance and chemoresistance traits. A549 cells (5000 cells per well) were seeded in a 96-well plate and incubated at 37°C with 5% CO2. After 24 hours, LV-NC and LV-TAGAP CD4 T cells were added to the wells at ratios of 1:2, 1:3, and 1:5. The co-cultures were incubated for 24 hours, and the absorbance of each well was measured at 490 nm using an LDH cytotoxicity assay kit.

### 2.10 Chemotaxis assay

A 5.0-μm pore size Transwell chamber was used for the experiment. 1×10^5^ A549 cells in 600 μL of medium were added to the lower chamber, while 5×10^5^ CD4 T cells in 100 μL of medium were added to the upper chamber. The chambers were incubated at 37°C for 24 hours. After incubation, the CD4 T cell suspension in the upper chamber was aspirated, and the remaining cells in the upper chamber were counted using a cell counting plate. The migrated cell number was determined, and the migration rate was analyzed.

### 2.11 Western blot analysis

In brief, protein extraction, quantification, and isolation were performed using 10% SDS-PAGE. Subsequently, the protein bands were transferred onto PVDF membranes (Bio-Rad, CA, USA). After blocking with 4% skim milk powder, the membranes were incubated overnight at 4°C with primary antibodies, followed by incubation with secondary antibodies (diluted 1:5000, Cell Signaling Technology, Beverly, MA, USA) for 2 hours. Finally, chemiluminescence was used for band detection. The antibody information used in the Western blot is provided in the **Table 1** below.

**Table 1:** Antibodies Used in the Study, Dilution Ratios, Manufacturers, and Species.

Antibody	Dilution Ratio	Manufacturer	Species
GAPDH (primary)	1:5000	Huabio	Rabbit
JAK1 (primary)	1:1000	ABclonal	Rabbit
pJAK1 (primary)	1:1000	ABclonal	Rabbit
JAK2 (primary)	1:1000	ABclonal	Rabbit
pJAK2 (primary)	1:1000	ABclonal	Rabbit
STAT1 (primary)	1:1000	ABclonal	Rabbit
pSTAT1 (primary)	1:1000	ABclonal	Rabbit
STAT3 (primary)	1:1000	ABclonal	Rabbit
pSTAT3 (primary)	1:1000	ABclonal	Rabbit
IgG (secondary)	1:2000	Beyotime	Goat anti-rabbit

### 2.12 *In vivo* experiment

A total of 12 female NSG mice aged 4-5 weeks (Shanghai Southern Model Biological Technology Co., Ltd., China) were used to establish a subcutaneous tumor model of lung adenocarcinoma A549. Tumor volumes were determined using caliper measurements and calculated using the formula: V = 0.5 × L × W^2, where V represents the tumor volume, L indicates the longest diameter, and W stands for the width at the widest point perpendicular to the length. When the tumor volume reached 100 mm^3^, the mice were randomly divided into two groups. Stable TAGAP-expressing CD4+ T cells were intravenously injected into the mice, with each mouse receiving 1×10^7^ CD4+ T cells. A control group was also included. Tumor volume was measured every three days, and the changes in volume were recorded to plot the tumor growth curve. After 30 days, the mice were euthanized, and the tumors were excised, photographed, and preserved in formalin for subsequent pathological experiments.

### 2.13 Immunohistochemistry

The anti-TAGAP (1:100) and anti-CD8/CD4 (1:100) were purchased from Solarbio-Beijing and ABclonal.The goat anti-rabbit secondary antibody was purchased from Beyotime Institute of Biotechnology.

Fixed tumor tissues were dehydrated, embedded in paraffin, and sliced into 4 μm sections. The sections were deparaffinized in two xylene baths for 20 minutes each, followed by hydration in a series of graded ethanol baths for 5 minutes each. Antigen retrieval was performed using EDTA antigen retrieval solution for 20 minutes. After PBS washing, the sections were incubated with a peroxidase-blocking reagent at room temperature for 10 minutes to block endogenous peroxidase activity. The sections were then blocked with normal goat serum for 30 minutes. CD4 antibody (ABCAM, USA) was added to the tissue sections and incubated overnight at 4°C. Afterward, the sections were incubated with HRP-conjugated goat anti-rabbit secondary antibody (Beyotime, China) at 37°C for 30 minutes, followed by 5-minute DAB staining. After PBS washing, the sections were counterstained with hematoxylin, dehydrated in graded ethanol and xylene, and mounted. Photographic documentation and analysis of the staining results were performed using an upright light microscope (Leica DM 4000B, Germany).

### 2.14 ELISA

The harvested tumor tissues were ground, and the expression levels of CD4+ T cell-related cytokines were measured using an ELISA kit according to the manufacturer’s instructions (Elabscience, China). The optical density (OD) values were measured at a wavelength of 450 nm using an ELISA reader.

### 2.15 Statistical analysis

Pearson or Spearman correlation coefficients were used to assess the correlation between TAGAP mRNA expression and the various categories of scores in the LUAD dataset. Differences in expression between normal and malignant samples were calculated for each tumor using the Wilcoxon test. p<0.05 was considered to be statistically significant.

## 3 Results

### 3.1 Expression level and prognostic significance of TAGAP in LUAD

**Figure 1:**
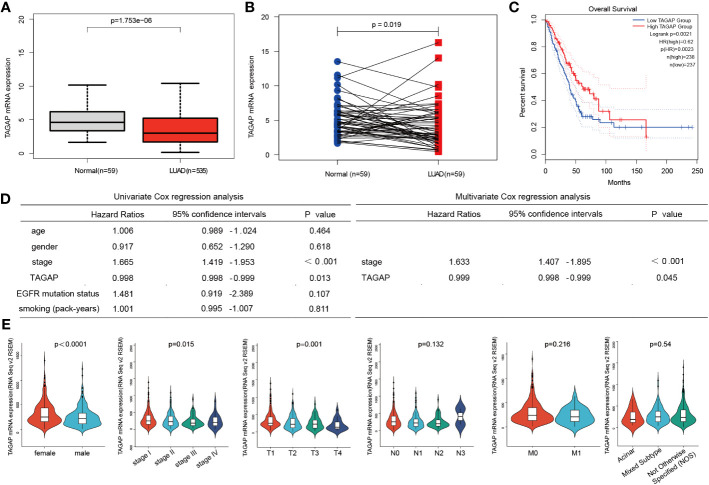
Expression and prognostic role of TAGAP in LUAD. **(A, B)** Paired and unpaired differential expressions of TAGAP in LUAD and normal tissues. **(C)** TAGAP’s KM survival analysis curve in LUAD. **(D)** Univariate and multivariate regression analyses of TAGAP and LUAD’s clinicopathological characteristics. **(E)** Association between TAGAP expression and clinicopathologic characteristics.

We analyzed the TCGA-LUAD dataset (59 normal and 535 LUAD samples). A significantly low expression was observed in LUAD tissues compared to normal ones, similar to LUAD and paired paracancerous tissues (**Figures 1A, B**). The GEPIA survival analysis indicated that a higher TAGAP expression was related to a more favorable prognosis in LUAD patients (**Figure 1C**). Additionally, after excluding incomplete data, we analyzed the correlation between TCGAP and clinical data in 474 TCGA-LUAD samples. Univariate Cox regression analysis and multivariate Cox regression analysis showed that TAGAP expression and tumor stage were risk factors for overall survival in LUAD and were independent prognostic factors (**Figure 1D**). Furthermore, TAGAP mRNA expression levels in LUAD were significantly correlated with gender (p<0.001), pathological stage (p =0.015), and T stage(p =0.001), but not with N stage(p =0.132), M stage(p =0.216), or pathological subtype(p =0.54) (**Figure 1E**).

### 3.2 Correlation of TAGAP expression levels with immune cell infiltration in LUAD

**Figure 2:**
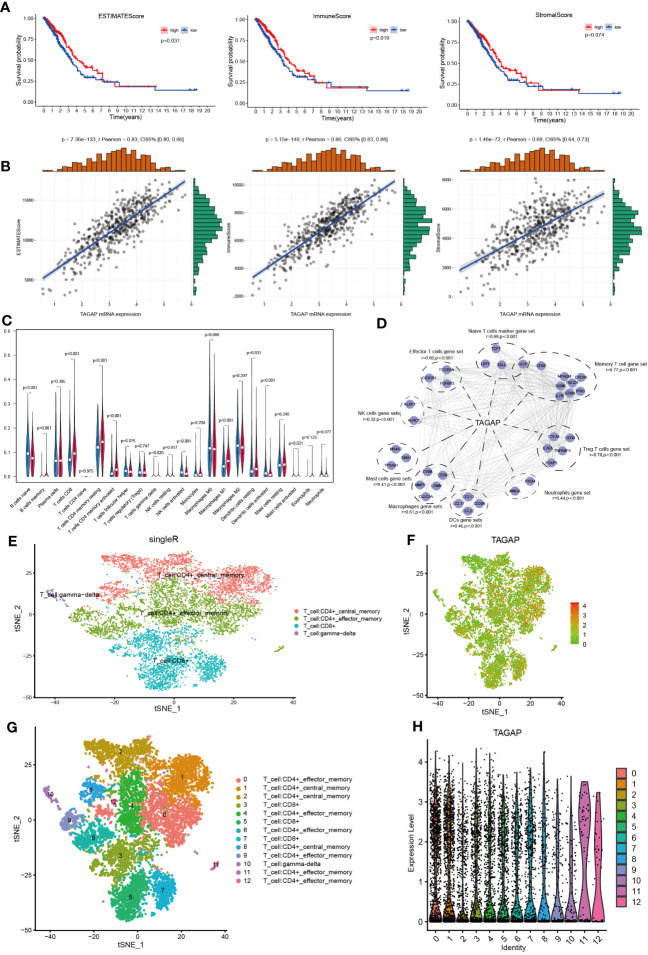
Correlation analysis of TAGAP and immune cell infiltration in LUAD. **(A)** Survival analyses of high- and low- ESTIMATE, immune, and stromal scores groups in LUAD. **(B)** Correlation analyses between TAGAP and ESTIMATE, immune, and stromal scores in LUAD. **(C)** Analyses of the difference in immune cell infiltration between the high and low TAGAP expression groups in LUAD by CIBERSORT. **(D)** Correlation analyses between TAGAP expression and different types of immune cell signatures in LUAD by GEPIA. **(E, G)** Cells were annotated in 4 and 12 clusters. **(F, H)** Expression and distribution of TAGAP in 4 and 12 clusters of immune cells.

According to previous immune-related research, TAGAP is primarily engaged in the activation of antifungal signaling pathways in macrophages and DC, as well as directing T-helper cell differentiation and regulating thymocyte formation (11, 19). Given the known immune functions of TAGAP, it could be hypothesized that its expression might be associated with the immune landscape of LUAD. Using ESTIMATE, we first determined the stromal, immune, and ESTIMATE scores for each TCGA-LUAD tumor sample. These scores were then aggregated by median to depict the K-M survival analysis. According to these findings, high immune and ESTIMATE scores were linked to a positive LUAD prognosis, whereas the stromal score did not affect prognosis (**Figure 2A**). Then, we calculated the relationship between TAGAP expression and the abovementioned results. The TAGAP expression had a strong positive connection with stromal, immune, and ESTIMATE scores (**Figure 2B**).

To further explore the correlation between TAGAP and different immune cells, we used the CIBERSORT algorithm to infer the content of 22 immune cells in each LUAD sample. Results showed clear differences in the distribution of immune cell subpopulations in the TAGAP high and low expression groups. In the TAGAP high expression group, the expression of T cell CD8, T cell CD4 memory resting, T-cell CD4 memory activation, macrophage M1, and dendritic cells resting were significantly higher (**Figure 2C**).

Then, GEPIA was used to evaluate the association between TAGAP expression and immune cell signature genes of different tumor invasion types. The GEPIA correlation analysis results confirmed that, in LUAD, TAGAP expression was correlated with marker gene sets of different immune cells in different degrees. The gene markers affected by TAGAP expression included Naive T cells, Effector T cells, Memory T cells, Treg T cells, Neutrophils, Mast cells, NK cells, DCs, and Macrophages gene sets. TAGAP showed a significant positive correlation with all of the above gene sets (**Figure 2D**).

To validate the association between TAGAP and tumor-infiltrating lymphocytes, we analyzed publicly available single-cell RNA sequencing datasets of lung adenocarcinoma. After quality control and normalization, t-distributed stochastic neighbor embedding (tSNE) was performed on peripheral cells (PCs) to cluster cell populations. Subsequent immunophenotyping revealed an enriched expression of TAGAP in specific clusters. It’s important to note that similar names across different clusters were used to denote cells of similar type or state. For example, the same names in **Figures 2E, G** represent similar cellular populations, albeit the different number of clusters resulted from the level of granularity in the analysis. Immunophenotyping revealed enriched expression of TAGAP in CD4+ and CD8+ T cell clusters (**Figures 2E-H**).

### 3.3 TAGAP expression levels can predict immunotherapy responsiveness

**Figure 3:**
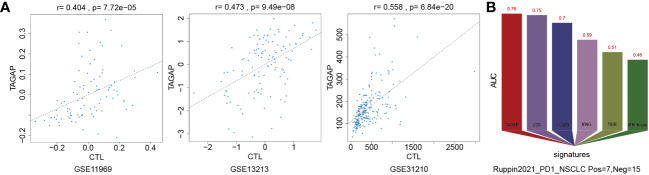
TAGAP expression correlates with CTLs and immunotherapy response in NSCLC. **(A)** Correlation analysis of TAGAP expression and cytotoxic T lymphocytes (CTL) in GSE11969, GSE13213 and GSE31210 by TIDE **(B)** TIDE biomarker evaluation of TAGAP response to immunotherapy in NSCLC.

We used the Tumor Immune Dysfunction and Exclusion (TIDE) framework to confirm TAGAP’s relevance to immune cells and determine whether it has predictive value for immunotherapy. Pearson correlation analysis was used to quantify the expression of TAGAP and the level of cytotoxic T lymphocytes (CTL) in GSE11969, GSE13213, and GSE31210. TAGAP expression levels and CTL were shown to be significantly correlated in above three GEO-LUAD datasets (**Figure 3A**). We compared the area under the ROC curve (AUC) for TAGAP expression to other biomarker signatures, such as CD8, CD274 (PD-L1), interferon gamma (IFNG), TIDE, and microsatellite instability (MSI) score, as a tool for predicting immunotherapy response. TAGAP had the greatest predictive performance in a NSCLC (20) cohort (Ruppin_PD1_NSCLC, positive=7, negative =15), comparable to CD8. TAGAP, CD8, and CD274 all had an AUC greater than 0.7, indicating a significant probability of a positive immunotherapy response (**Figure 3B**).

### 3.4 IHC and RT-qPCR experimental validation of TAGAP expression increases

**Figure 4:**
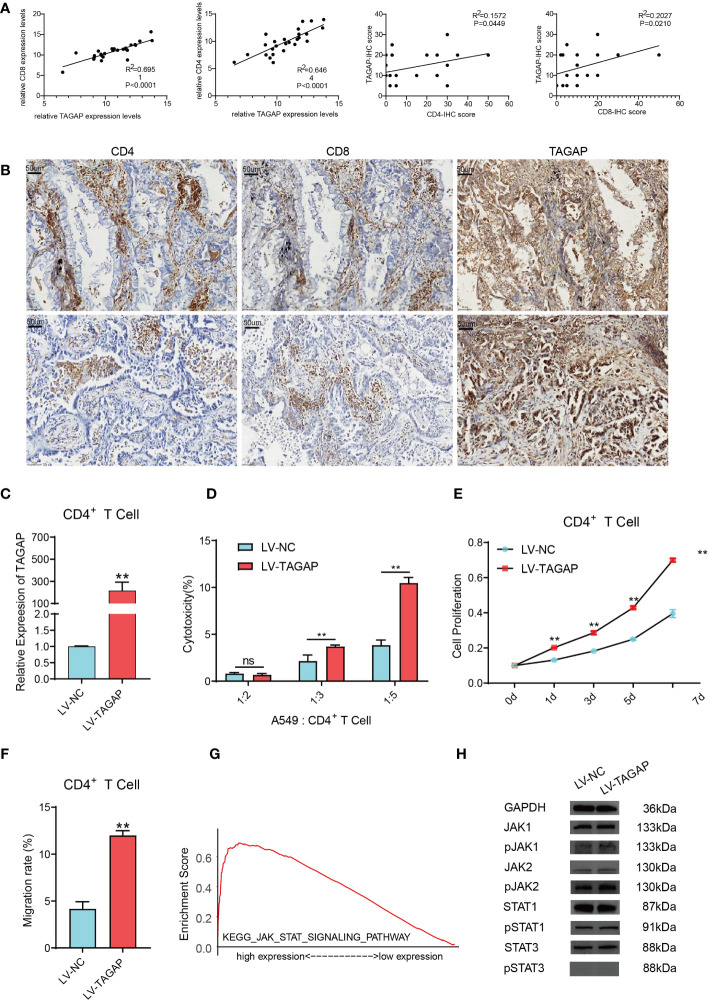
TAGAP enhances the cytotoxicity and chemotaxis of CD4+ T cells and activates the STAT pathway. **(A)** RT-qPCR and immunohistochemical correlation analyses of TAGAP with CD8 and CD4 in 26 pairs of LUAD tissues. **(B)** Representative images of CD8, CD4, and TAGAP immunohistochemistry in 26 pairs of LUAD tissues. **(C)** TAGAP expression in CD4+ T cells was detected; **(D)** LDH assay was used to determine the cytotoxicity of CD4+ T cells; **(E)** MTT assay was performed to assess the proliferation capacity of CD4+ T cells; **(F)** Transwell assay was conducted to measure the chemotaxis of CD4+ T cells. **(G)** GSEA pathway enrichment analysis was performed; **(H)** Expression of JAK/STAT pathway-related proteins was detected; ** p<0.01; ns, not significant.

The expression of TAGAP, CD8, and CD4 was detected in 26 LUAD tissues (**Figures 4A, B**). Results showed a significant positive correlation between TAGAP and the expression of CD8 (R^2 = ^0.6951, *p* < 0.0001) and CD4 (R^2 = ^0.6464, *p* < 0.0001). Additionally, we collected tumor tissues from 26 LUAD patients and performed immunohistochemical experiments to detect the proportion of positive cells for CD8 and CD4 surface antigen, and TAGAP in immune infiltrating cells. Results showed that the expression of TAGAP in immune cells was significantly and positively correlated with the expression of CD8 (R^2 = ^0.2027, *p* = 0.021) and CD4 (R^2 = ^0.1572, *p* = 0.0449). Altogether, these results suggested that TAGAP expression was also higher in tissues with more infiltration of CD8+ and CD4+ T cells.

### 3.5 Enhancement of CD4+ T cell chemotaxis and cytotoxicity by TAGAP

Although TAGAP expression showed a stronger correlation with CD8+ T cell infiltration, we focused on the impact of TAGAP on CD4+ T cell function in this study. CD4+ T cells play a central role in regulating anti-tumor immune responses by secreting cytokines to activate other immune cells such as CD8+ T cells and macrophages. Additionally, subsets of CD4+ T cells also combat tumors through various mechanisms including direct killing of tumor cells and regulation of inflammation within the microenvironment. Therefore, we sought to investigate whether TAGAP could modulate CD4+ T cell activity, which would imply an important role of TAGAP in orchestrating anti-tumor immunity. To investigate the impact of TAGAP on CD4+ T cell function, we first transfected CD4+ T cells with a lentiviral vector overexpressing TAGAP. The expression of TAGAP in CD4+ T cells was examined using qRT-PCR. The results showed a significant upregulation of TAGAP expression in CD4+ T cells of the LV-TAGAP group compared to the control group (**Figure 4C**). Subsequently, to assess the effect of TAGAP on CD4+ T cell cytotoxicity, we co-cultured tumor cells with activated CD4+ T cells at different target-to-effector cell (T:E) ratios of 1:2, 1:3, and 1:5. The cytotoxicity of CD4+ T cells was determined using an LDH assay. The results demonstrated that overexpression of TAGAP significantly enhanced the cytotoxicity of CD4+ T cells against lung adenocarcinoma cells compared to the control group, with the highest cytotoxicity observed at the T:E ratio of 1:5 (**Figure 4D**). Additionally, the effect of TAGAP on the proliferation of CD4+ T cells was evaluated using the MTT assay. The results revealed that overexpression of TAGAP significantly promoted the proliferation capacity of CD4+ T cells compared to the control group (**Figure 4E**). Lastly, a chemotaxis assay was performed to examine the impact of TAGAP on the chemotaxis of CD4+ T cells. The results showed that overexpression of TAGAP significantly enhanced the chemotaxis ability of CD4+ T cells compared to the control group (**Figure 4F**).

### 3.6 Activation of the STAT pathway by TAGAP overexpression

To further elucidate the specific molecular mechanisms through which TAGAP regulates CD4+ T cell function, we performed Gene Set Enrichment Analysis (GSEA) on TAGAP. The results revealed a significant enrichment of TAGAP in the JAK-STAT signaling pathway (**Figure 4G**). Subsequently, we examined the expression of JAK-STAT pathway-related proteins (JAK1, JAK2, STAT1, STAT3, p-JAK1, p-JAK2, p-STAT1, p-STAT3) in CD4+ T cells overexpressing TAGAP. The results demonstrated that TAGAP overexpression activated the STAT pathway signaling (**Figure 4H**).

### 3.7 *In vivo* validation of the impact of TAGAP on CD4+ T cell cytotoxicity

**Figure 5:**
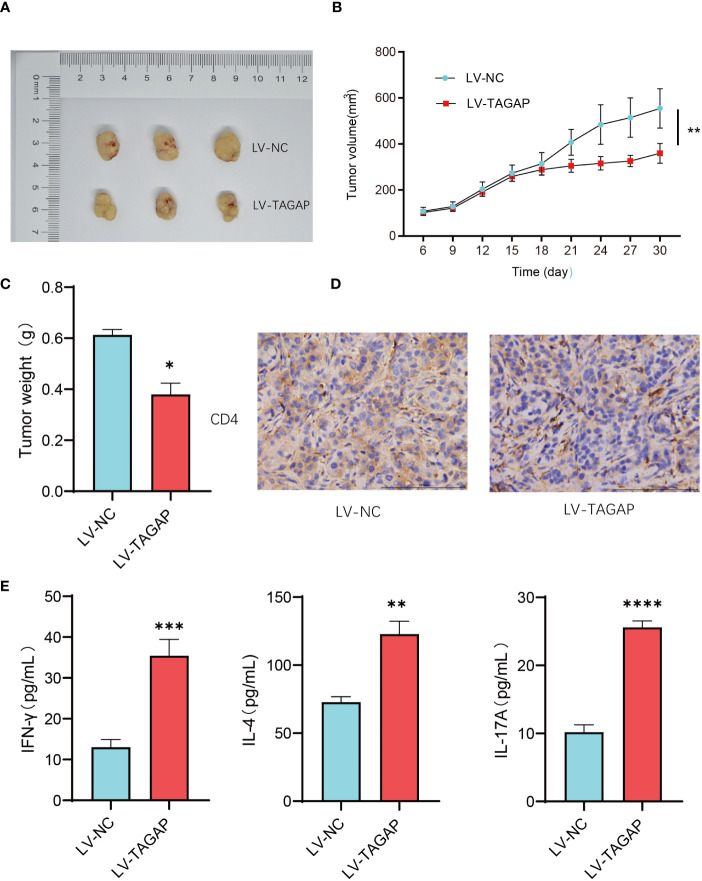
The effect of TAGAP on CD4+ T cell cytotoxicity was validated in a mouse xenograft tumor model. **(A)** Tumor size after different treatments; **(B, C)** Tumor volume and weight were measured in different treatment groups; **(D)** Immunohistochemistry staining was used to detect the expression of CD4; **(E)** Expression of cytokines IFN-γ, IL-4, and IL-17A secreted by CD4+ T cells was assessed. * p < 0.05, ** p < 0.01, *** p <0.001, **** p <0.0001.

To validate the findings from the *in vitro* cell experiments, we utilized B-NSG severely immunodeficient mice and established a xenograft tumor model by subcutaneously injecting A549 cells. Subsequently, LV-NC and LV-TAGAP CD4+ T cells were intravenously injected via the tail vein. Tumor growth was monitored, and it was observed that LV-TAGAP significantly suppressed tumor growth (**Figures 5A-C**). Immunohistochemistry (IHC) results demonstrated that LV-TAGAP significantly enhanced the expression of CD4 (**Figure 5D**). Additionally, LV-TAGAP increased the levels of the cytokines IFN-γ, IL-4, and IL-17A secreted by CD4+ T cells (**Figure 5E**). Collectively, these findings suggest that TAGAP overexpression can promote the cytotoxicity of CD4+ T cells (**Figure 5E**).

## 4 Discussion

With the recent advancements in immunotherapy, there has been increasing interest in the activation, differentiation, and function of immune cells and their relationship to the efficacy of immunotherapy (21). However, the majority of research has focused on CD8+ T cells, NK cells, and macrophages, while CD4+ T cells have received less attention due to their complex immunoregulatory roles (22–24). Our study presents novel insights into the critical role of TAGAP in Lung Adenocarcinoma (LUAD), emphasizing its prognostic potential, relationship with immune cell infiltration, influence on CD4+ T cell activity, and predictive value for immunotherapy responsiveness.

TAGAP is a T cell Rho-GTPase activating protein that is expressed when T cells are activated. In granulocytes, TAGAP induction in response to Mycobacterium tuberculosis infection involves enrichment of differential acetylation (DA) peaks (25). Moreover, dectin-induced signaling was linked to the activation of an effective T helper cell immune response during antifungal host defense and autoimmunity, with TAGAP participation (11). On the other hand, the function of TAGAP in cancer is currently unknown.

In the present study, we confirmed that TAGAP can be considered as a gene associated with the tumor microenvironment and LUAD’s overall survival. TAGAP is a novel biomarker associated with immune scoring in LUAD, and its low expression independently predicts poor outcomes in LUAD patients. Additionally, we found that different immunomarker groups and immune infiltration levels were positively correlated with TAGAP expression in LUAD. Previous studies have demonstrated that the tumor microenvironment can promote tumor progression and immune response through inflammation, angiogenesis, immunomodulatory, and therapeutic responses (26). Understanding the molecular composition and function of the tumor microenvironment is critical for the development of LUAD therapies targeting angiogenesis and recent immune checkpoints.

We extended our investigations to explore the influence of TAGAP on CD4+ T cell function, which is pivotal in orchestrating anti-tumor immune responses. The results revealed that TAGAP overexpression enhanced CD4+ T cell cytotoxicity and chemotaxis, bolstering the proliferation capacity of these cells. The activation of the JAK-STAT signaling pathway by TAGAP overexpression offers a plausible molecular mechanism underlying TAGAP’s impact on CD4+ T cell function, warranting further investigations. The JAK/STAT pathway is the main signal transduction mechanism of various cytokines and growth factors (27, 28). Its role in lung cancer has been confirmed by several previous studies. These results further supported the hypothesis that TAGAP plays a reliable role in the occurrence and development of LUAD.

Although studies have shown the relationship between TAGAP and the prognosis of LUAD patients, the detailed mechanism and immunoclustering distribution of TAGAP in NSCLC is still unclear. In the current study, the results of analysis of lung cancer samples from the GEO single-cell dataset, RT-qPCR, and immunohistochemical analysis showed that T CD4+/CD8+ cell expression was correlated to TAGAP. A previous study showed that cytokines released by T cells can influence the activation of STATs (29). We speculated that the regulation of TAGAP on the immune microenvironment, especially on T cells, can affect the release of cytokines, and ultimately promote the occurrence and development of tumor cells through the JAK-STAT pathway.

TAGAP has been discovered to have crucial effects through additional pathways and systems, in addition to studies in the tumor immune microenvironment. TAGAP-deficient macrophages had enhanced viral replication of herpes simplex virus type 1, lower expression of pro-inflammatory chemokines and cytokines, and lower production of IFN-β, according to Juan Liao et al., implying that TAGAP plays a significant role in antiviral cytokine production (30). TAGAP governs the cytoskeletal architecture of actin fibers via RhoA, which is required for cell contractility and the cycle of integrin attachment and separation required for directional migration of thymocytes, according to Jonathan S. Duke-Cohan et al (19). Given the widespread link of TAGAP with a variety of disorders, further research is needed to figure out how TAGAP works.

In terms of immunotherapy responsiveness, our study suggests that TAGAP expression levels can predict immunotherapy efficacy, with TAGAP outperforming established biomarkers such as PD-L1 in certain contexts. Finally, our *in vivo* and *in vitro* validation further strengthened the hypothesis of TAGAP’s role in enhancing CD4+ T cell cytotoxicity. These results suggest that TAGAP could be an effective therapeutic target to improve the antitumor immune response in LUAD, providing a novel angle for future research.

Altogether, our findings shed light on the multifaceted role of TAGAP in LUAD, emphasizing its prognostic and therapeutic potential. Further studies are warranted to fully elucidate the underlying mechanisms of TAGAP’s interaction with the tumor microenvironment and its potential clinical applications.

## Data availability statement

The original contributions presented in the study are included in the article/supplementary material. Further inquiries can be directed to the corresponding author.

## Ethics statement

Ethical approval was not required for the studies on humans in accordance with the local legislation and institutional requirements because only commercially available established cell lines were used. The animal study was approved by Animal Ethics Committee of the First Affiliated Hospital of Guangxi Medical University. The study was conducted in accordance with the local legislation and institutional requirements.

## Author contributions

ZX and ZZ devised and developed the experiments, carried out the RT-qPCR and IHC tests, created figures and/or tables, and approved the final document. ZZ, WJ, and LH evaluated bioinformatics data, prepared or reviewed drafts of the publication, and approved the final draft. Figures and/or tables were developed by YS, TZ, FQ, and JQ, who also approved the final draft. The experiments were devised and designed by SL, who also authorized the final draft. All authors contributed to the article and approved the submitted version.

## References

[1] Sung H Ferlay J Siegel RL Laversanne M Soerjomataram I Jemal A. Global cancer statistics 2020: GLOBOCAN estimates of incidence and mortality worldwide for 36 cancers in 185 countries. CA Cancer J Clin (2021) 71(3):209–49. doi: 10.3322/caac.21660 33538338

[2] Dalwadi SM Lewis GD Bernicker EH Butler EB Teh BS Farach AM. Disparities in the treatment and outcome of stage I non-small-cell lung cancer in the 21st century. Clin Lung Cancer (2019) 20(3):194–200. doi: 10.1016/j.cllc.2018.11.004 30655194

[3] Kita T Araya T Sakai T Uchida Y Matsuoka H Kasahara K. Nivolumab-induced polymyalgia rheumatica in a patient with lung adenocarcinoma. Am J Med Sci (2021) 362(3):321–3. doi: 10.1016/j.amjms.2021.04.010 33905737

[4] Reck M Remon J Hellmann MD. First-line immunotherapy for non-small-cell lung cancer. J Clin Oncol (2022) 40(6):586–97. doi: 10.1200/JCO.21.01497 34985920

[5] Niknafs N Balan A Cherry C Hummelink K Monkhorst K Shao XM. Persistent mutation burden drives sustained anti-tumor immune responses. Nat Med (2023) 29(2):440–9. doi: 10.1038/s41591-022-02163-w PMC9941047 36702947

[6] Liu S Meng Y Liu L Lv Y Yu W Liu T. CD4(+) T cells are required to improve the efficacy of CIK therapy in non-small cell lung cancer. Cell Death Dis (2022) 13(5):441. doi: 10.1038/s41419-022-04882-x 35523765 PMC9076680

[7] Wang L Wu W Zhu X Ng W Gong C Yao C. The ancient chinese decoction Yu-Ping-Feng suppresses orthotopic lewis lung cancer tumor growth through increasing M1 macrophage polarization and CD4(+) T cell cytotoxicity. Front Pharmacol (2019) 10:1333. doi: 10.3389/fphar.2019.01333 31780946 PMC6857089

[8] Chao JL Savage PA. Unlocking the complexities of tumor-associated regulatory T cells. J Immunol (2018) 200(2):415–21. doi: 10.4049/jimmunol.1701188 PMC5763514 29311383

[9] Tamehiro N Nishida K Yanobu-Takanashi R Goto M Okamura T Suzuki H. T-cell activation RhoGTPase-activating protein plays an important role in T(H)17-cell differentiation. Immunol Cell Biol (2017) 95(8):729–35. doi: 10.1038/icb.2017.27 28462950

[10] Saoudi A Kassem S Dejean A Gaud G. Rho-GTPases as key regulators of T lymphocyte biology. Small GTPases (2014) 5:e28208. doi: 10.4161/sgtp.28208 24825161 PMC4160340

[11] Chen J He R Sun W Gao R Peng Q Zhu L. TAGAP instructs Th17 differentiation by bridging Dectin activation to EPHB2 signaling in innate antifungal response. Nat Commun (2020) 11(1):1913. doi: 10.1038/s41467-020-15564-7 32312989 PMC7171161

[12] Arshad M Bhatti A John P Jalil F Borghese F Kawalkowska JZ. T cell activation Rho GTPase activating protein (TAGAP) is upregulated in clinical and experimental arthritis. Cytokine (2018) 104:130–5. doi: 10.1016/j.cyto.2017.10.002 29017772

[13] Wang Z Xu H Zhu L He T Lv W Wu Z. Establishment and evaluation of a 6-gene survival risk assessment model related to lung adenocarcinoma microenvironment. BioMed Res Int (2020) 2020:6472153. doi: 10.1155/2020/6472153 32337264 PMC7157809

[14] Chang JT Lee YM Huang RS. The impact of the Cancer Genome Atlas on lung cancer. Transl Res (2015) 166(6):568–85. doi: 10.1016/j.trsl.2015.08.001 PMC4656061 26318634

[15] Ritchie ME Phipson B Wu D Hu Y Law CW Shi W. limma powers differential expression analyses for RNA-sequencing and microarray studies. Nucleic Acids Res (2015) 43(7):e47. doi: 10.1093/nar/gkv007 25605792 PMC4402510

[16] Tang Z Li C Kang B Gao G Li C Zhang Z. GEPIA: a web server for cancer and normal gene expression profiling and interactive analyses. Nucleic Acids Res (2017) 45(W1):W98–98W102. doi: 10.1093/nar/gkx247 28407145 PMC5570223

[17] Fu J Li K Zhang W Wan C Zhang J Jiang P. Large-scale public data reuse to model immunotherapy response and resistance. Genome Med (2020) 12(1):21. doi: 10.1186/s13073-020-0721-z 32102694 PMC7045518

[18] Subramanian A Tamayo P Mootha VK Mukherjee S Ebert BL Gillette MA. Gene set enrichment analysis: a knowledge-based approach for interpreting genome-wide expression profiles. Proc Natl Acad Sci U.S.A. (2005) 102(43):15545–50. doi: 10.1073/pnas.0506580102 PMC1239896 16199517

[19] Duke-Cohan JS Ishikawa Y Yoshizawa A Choi YI Lee CN Acuto O. Regulation of thymocyte trafficking by Tagap, a GAP domain protein linked to human autoimmunity. Sci Signal (2018) 11(534):eaan8799. doi: 10.1126/scisignal.aan8799 29895617 PMC6393939

[20] Lee JS Ruppin E. Multiomics prediction of response rates to therapies to inhibit programmed cell death 1 and programmed cell death 1 ligand 1. JAMA Oncol (2019) 5(11):1614–8. doi: 10.1001/jamaoncol.2019.2311 PMC6707018 31436822

[21] Sharma P Allison JP. Immune checkpoint targeting in cancer therapy: toward combination strategies with curative potential. Cell (2015) 161(2):205–14. doi: 10.1016/j.cell.2015.03.030 PMC5905674 25860605

[22] Geginat J Paroni M Facciotti F Gruarin P Kastirr I Caprioli F. The CD4-centered universe of human T cell subsets. Semin Immunol (2013) 25(4):252–62. doi: 10.1016/j.smim.2013.10.012 24183700

[23] Zhu J Yamane H Paul WE. Differentiation of effector CD4 T cell populations (*). Annu Rev Immunol (2010) 28:445–89. doi: 10.1146/annurev-immunol-030409-101212 PMC3502616 20192806

[24] Kather JN Suarez-Carmona M Charoentong P Weis CA Hirsch D Bankhead P. Topography of cancer-associated immune cells in human solid tumors. Elife (2018) 7:e36967. doi: 10.7554/eLife.36967 30179157 PMC6133554

[25] Sivakumaran D Blatner G Bakken R Hokey D Ritz C Jenum S. A 2-dose AERAS-402 regimen boosts CD8(+) polyfunctionality in HIV-negative, BCG-vaccinated recipients. Front Immunol (2021) 12:673532. doi: 10.3389/fimmu.2021.673532 34177914 PMC8231292

[26] Altorki NK Markowitz GJ Gao D Port JL Saxena A Stiles B. The lung microenvironment: an important regulator of tumour growth and metastasis. Nat Rev Cancer (2019) 19(1):9–31. doi: 10.1038/s41568-018-0081-9 30532012 PMC6749995

[27] Pastuszak-Lewandoska D Domańska-Senderowska D Kordiak J Antczak A Czarnecka KH Migdalska-Sęk M. Immunoexpression analysis of selected JAK/STAT pathway molecules in patients with non- small-cell lung cancer. Pol Arch Intern Med (2017) 127(11):758–64. doi: 10.20452/pamw.4115 28972958

[28] Sánchez-Ceja SG Reyes-Maldonado E Vázquez-Manríquez ME López-Luna JJ Belmont A Gutiérrez-Castellanos S. Differential expression of STAT5 and Bcl-xL, and high expression of Neu and STAT3 in non-small-cell lung carcinoma. Lung Cancer (2006) 54(2):163–8. doi: 10.1016/j.lungcan.2006.07.012 16959370

[29] Kryczek I Lin Y Nagarsheth N Peng D Zhao L Zhao E. IL-22(+)CD4(+) T cells promote colorectal cancer stemness via STAT3 transcription factor activation and induction of the methyltransferase DOT1L. Immunity (2014) 40(5):772–84. doi: 10.1016/j.immuni.2014.03.010 PMC4032366 24816405

[30] Liao J Jijon HB Kim IR Goel G Doan A Sokol H. An image-based genetic assay identifies genes in T1D susceptibility loci controlling cellular antiviral immunity in mouse. PloS One (2014) 9(9):e108777. doi: 10.1371/journal.pone.0108777 25268627 PMC4182575

